# Tetra-*n*-butyl­ammonium tricyanido[*N*-(2-pyridyl­carbon­yl)pyridine-2-carbox­imidato]ferrate(III) dihydrate

**DOI:** 10.1107/S1600536811049415

**Published:** 2011-11-30

**Authors:** Yunyun Xiao, Xiaoping Shen, Yizhi Li

**Affiliations:** a School of Chemistry and Chemical Engineering, Jiangsu University, Zhenjiang 212013, People’s Republic of China; b State Key Laboratory of Coordination Chemistry, Nanjing University, Nanjing 210093, People’s Republic of China

## Abstract

In the title compound, (C_16_H_36_N)[Fe(C_12_H_8_N_3_O_2_)(CN)_3_]·2H_2_O, the tetra-*n*-butyl­ammonium ion has a tetra­hedral configuration around the N atom, while the Fe^III^ atom of the tricyanido[*N*-(2-pyridyl­carbon­yl)pyridine-2-carboximidato]iron(III) anion adopts a distorted octa­hedral geometry. O—H⋯O and O—H⋯N hydrogen bonds link the components in the crystal structure.

## Related literature

For related structures of the [Fe(bpca)(CN)_3_]^−^ anion (bpca is bis(2-pyridylcarbonyl)amidate) with different cations, see: Lescouëzec *et al.* (2004 ▶); Ouahab *et al.* (2005 ▶). For related cyanido-bridged complexes with [Fe(bpca)(CN)_3_]^−^ as a building block, see: Lescouëzec *et al.* (2004 ▶); Wen *et al.* (2006 ▶).
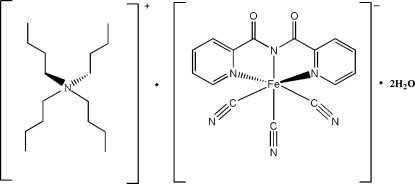


## Experimental

### 

#### Crystal data

(C_16_H_36_N)[Fe(C_12_H_8_N_3_O_2_)(CN)_3_]·2H_2_O
*M* *_r_* = 638.61
Monoclinic, 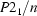

*a* = 13.142 (2) Å
*b* = 15.663 (3) Å
*c* = 17.097 (3) Å
β = 90.48 (3)°
*V* = 3519.0 (11) Å^3^
*Z* = 4
Mo *K*α radiation
μ = 0.47 mm^−1^
*T* = 173 K
0.21 × 0.16 × 0.12 mm

#### Data collection

Rigaku Saturn 724 CCD diffractometer
Absorption correction: multi-scan (*ABSCOR*; Higashi, 1995 ▶) *T*_min_ = 0.887, *T*_max_ = 0.904
17432 measured reflections
6359 independent reflections
4300 reflections with *I* > 2σ(*I*)
*R*_int_ = 0.063

#### Refinement

*R*[*F* ^2^ > 2σ(*F* ^2^)] = 0.060
*wR*(*F* ^2^) = 0.135
*S* = 1.05
6359 reflections
392 parameters
6 restraints
H-atom parameters constrained
Δρ_max_ = 0.30 e Å^−3^
Δρ_min_ = −0.31 e Å^−3^

### 

Data collection: *CrystalClear* (Rigaku, 2008 ▶); cell refinement: *CrystalClear*; data reduction: *CrystalClear*; program(s) used to solve structure: *SHELXS97* (Sheldrick, 2008 ▶); program(s) used to refine structure: *SHELXS97* (Sheldrick, 2008 ▶); molecular graphics: *DIAMOND* (Brandenburg, 2006 ▶); software used to prepare material for publication: *SHELXTL* (Sheldrick, 2008 ▶).

## Supplementary Material

Crystal structure: contains datablock(s) I, global. DOI: 10.1107/S1600536811049415/zl2426sup1.cif

Structure factors: contains datablock(s) I. DOI: 10.1107/S1600536811049415/zl2426Isup2.hkl

Additional supplementary materials:  crystallographic information; 3D view; checkCIF report

## Figures and Tables

**Table 1:** Hydrogen-bond geometry (Å, °)

*D*—H⋯*A*	*D*—H	H⋯*A*	*D*⋯*A*	*D*—H⋯*A*
O1*W*—H1*X*⋯O1^i^	0.85	2.12	2.964 (10)	173
O1*W*—H1*Y*⋯O2*W*	0.85	2.25	3.058 (10)	158
O2*W*—H2*X*⋯N2^ii^	0.85	2.04	2.895 (10)	180
O2*W*—H2*Y*⋯N3	0.85	2.18	3.029 (11)	174

Symmetry codes: (i) 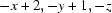; (ii) 
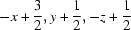
.

**Fig. 1.**
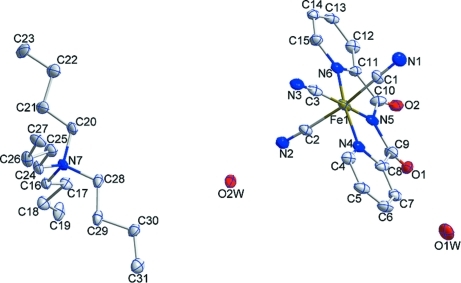
ORTEP diagram of the title complex with displacement ellipsoids drawn at the 30% probability level. Hydrogen atoms have been omitted for clarity.

**Fig. 2.**
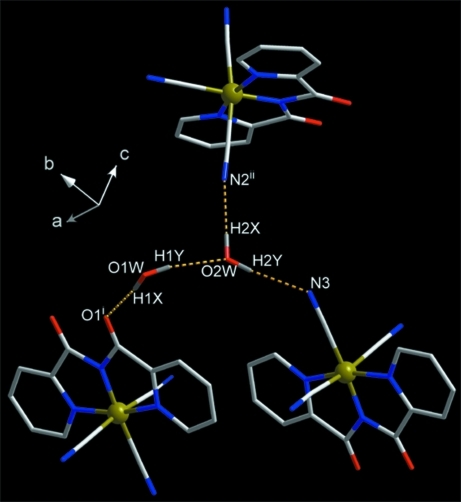
Packing diagram of the title complex, showing the hydrogen-bonding interactions. The [(n-C4H9)4N]+ cations have been omitted for clarity. Symmetry codes: (i) x + 1/2, -y + 1/2, z - 1/2; (ii) - x + 3/2, y - 1/2, -z + 1/2.

## supplementary crystallographic information

### Comment

[Fe(bpca)(CN)_3_]^-^ {bpca = [*N*-(2-pyridylcarbonyl) pyridine-2-carboximidate}, a low-spin iron^III^ complex with three cyanide ligands and a tridentate N-donor ligand around iron^III^ in a *mer* arrangement, is an interesting building block because it can not only coordinate to transition metal ions to form various polynuclear and one-dimensional structures with fascinating magnetic properties (Lescouëzec *et al.*, 2004; Wen *et al.*, 2006), but also combine with functionalized organic donors such as DIET and DIEDO (DIET = diiodoethylenedithotetrathiavalene and DIEDO = diiodoethylenedioxotetrathiavalene) to form charge transfer salts, which showed interesting electrical conducting and magnetic behaviors (Ouahab *et al.*, 2005). In a previous study, the crystal structure of the mononuclear complex PPh_4_[Fe^III^(bpca)(CN)_3_].H_2_O (PPh_4_ = tetraphenylphosphonium) has been reported by Lescouëzec and his coworkers (Lescouëzec *et al.*, 2004). Recently, we have synthesized the compound [(*n*-C_4_H_9_)_4_N][Fe^III^(bpca)(CN)_3_].2H_2_O, which is an analog of PPh_4_[Fe^III^(bpca)(CN)_3_].H_2_O with the same anion. Herein, the crystal structure of the obtained complex is presented.

The structure of the title compound is similar to that of PPh_4_[Fe^III^(bpca)(CN)_3_].H_2_O except with different cations. The asymmetric unit of the title complex consists of a [(*n*-C_4_H_9_)_4_N]^+^ cation, a [Fe^III^(bpca)(CN)_3_]^-^ anion and two H_2_O molecules (Fig. 1). As usual, the [(*n*-C_4_H_9_)_4_N]^+^ cation has a tetrahedral configuration around the N atom. In the [Fe^III^(bpca)(CN)_3_]^-^ anion, the Fe^III^ ion is coordinated by three carbon atoms of cyanide groups and three N-donors from bpca ligand in a *mer*-arrangement, which results in a distorted octahedral geometry. The Fe1—N(bpca) bond distances vary in the range of 1.735 (8)–1.959 (7) Å, which are close to those (1.893 (2)–1.959 (2) Å) found in the complex of PPh_4_[Fe^III^(bpca)(CN)_3_].H_2_O (Lescouëzec *et al.*, 2004). The Fe1—C(cyano) bond lengths (1.933 (10)–1.966 (10) Å) are also similar to those [1.937 (3)–1.951 (3) Å] reported for PPh_4_[Fe^III^(bpca)(CN)_3_].H_2_O.

There are some hydrogen-bonding interactions between water molecules, between water and ligand bpca, and between water and the N atom of cyano groups, which hold two adjacent [Fe^III^(bpca)(CN)_3_]^-^ together by H-bonds (Table 1, Fig. 2).

### Experimental

The complex of Bu_4_N[Fe^III^(bpca)(CN)_3_].H_2_O was prepared according to a literature method (Wen *et al.*, 2006). Then, 0.1 mmoL (62 mg) of Bu_4_N[Fe^III^(bpca)(CN)_3_].H_2_O was added to a MeCN/H_2_O [4/1(V/V), 20 ml] mixture with stirring, The resulting solution was filtered and the filtrate was left to allow slow evaporation in the dark at room temperature. Yellow block-shaped crystals of the title complex suitable for single-crystal X-ray diffraction were obtained after two weeks. Anal. Calc. for C_31_H_48_Fe_1_N_7_O_4_: C, 58.30; H, 7.58; N, 15.35; Fe, 8.75%. Found: C, 58.56; H, 7.73; N, 15.01; Fe, 8.98%.

### Refinement

All non-H atoms were refined with anisotropic thermal parameters. All H atoms from the ligand bpca and [(*n*-C_4_H_9_)_4_N]^+^ cation were calculated in idealized positions and included in the refinement in a riding mode with *U*_iso_ for H assigned as 1.2 or 1.5 times *U*_eq_ of the attached atoms. The H atoms bound to oxygen atoms from crystallized water molecules were located from difference maps, initially refined with O—H and H—H restraints (O—H = 0.850 (1) Å, H—H > 1.300 (1) Å), and then as riding, with *U*_iso_(H) = 1.2*U*_eq_(O).

### Crystal data

**Table:**

(C_16_H_36_N)[Fe(C_12_H_8_N_3_O_2_)(CN)_3_]·2H_2_O	*F*(000) = 1364
*M**_r_* = 638.61	*D*_x_ = 1.205 Mg m^−^^3^
Monoclinic, *P*2_1_/*n*	Mo *K*α radiation, λ = 0.71073 Å
Hall symbol: -P 2yn	Cell parameters from 4928 reflections
*a* = 13.142 (2) Å	θ = 2.1–24.8°
*b* = 15.663 (3) Å	µ = 0.47 mm^−^^1^
*c* = 17.097 (3) Å	*T* = 173 K
β = 90.48 (3)°	Block, yellow
*V* = 3519.0 (11) Å^3^	0.21 × 0.16 × 0.12 mm
*Z* = 4	

### Data collection

**Table:**

Rigaku Saturn 724 CCD diffractometer	6359 independent reflections
Radiation source: fine-focus sealed tube	4300 reflections with *I* > 2σ(*I*)
graphite	*R*_int_ = 0.063
φ and ω scans	θ_max_ = 25.3°, θ_min_ = 3.0°
Absorption correction: multi-scan (*ABSCOR*; Higashi, 1995)	*h* = −15→13
*T*_min_ = 0.887, *T*_max_ = 0.904	*k* = −13→18
17432 measured reflections	*l* = −12→20

### Refinement

**Table:**

Refinement on *F*^2^	Primary atom site location: structure-invariant direct methods
Least-squares matrix: full	Secondary atom site location: difference Fourier map
*R*[*F*^2^ > 2σ(*F*^2^)] = 0.060	Hydrogen site location: inferred from neighbouring sites
*wR*(*F*^2^) = 0.135	H-atom parameters constrained
*S* = 1.05	*w* = 1/[σ^2^(*F*_o_^2^) + (0.0648*P*)^2^ + 0.034*P*] where *P* = (*F*_o_^2^ + 2*F*_c_^2^)/3
6359 reflections	(Δ/σ)_max_ < 0.001
392 parameters	Δρ_max_ = 0.30 e Å^−^^3^
6 restraints	Δρ_min_ = −0.31 e Å^−^^3^

### Special details

**Table:**

Geometry. All e.s.d.'s (except the e.s.d. in the dihedral angle between two l.s. planes) are estimated using the full covariance matrix. The cell e.s.d.'s are taken into account individually in the estimation of e.s.d.'s in distances, angles and torsion angles; correlations between e.s.d.'s in cell parameters are only used when they are defined by crystal symmetry. An approximate (isotropic) treatment of cell e.s.d.'s is used for estimating e.s.d.'s involving l.s. planes.
Refinement. Refinement of *F*^2^ against ALL reflections. The weighted *R*-factor *wR* and goodness of fit *S* are based on *F*^2^, conventional *R*-factors *R* are based on *F*, with *F* set to zero for negative *F*^2^. The threshold expression of *F*^2^ > σ(*F*^2^) is used only for calculating *R*-factors(gt) *etc*. and is not relevant to the choice of reflections for refinement. *R*-factors based on *F*^2^ are statistically about twice as large as those based on *F*, and *R*- factors based on ALL data will be even larger.

### Fractional atomic coordinates and isotropic or equivalent isotropic displacement parameters (Å^2^)

**Table:**

	*x*	*y*	*z*	*U*_iso_*/*U*_eq_	
C1	0.7225 (7)	0.4358 (6)	−0.0451 (5)	0.041 (2)	
C2	0.7239 (7)	0.3334 (6)	0.1624 (6)	0.039 (2)	
C3	0.6909 (7)	0.4886 (6)	0.0945 (6)	0.043 (2)	
C4	0.9232 (7)	0.4724 (6)	0.0962 (6)	0.046 (2)	
H4	0.8835	0.5192	0.1139	0.055*	
C5	1.0283 (7)	0.4770 (6)	0.0993 (6)	0.050 (2)	
H5	1.0606	0.5270	0.1188	0.059*	
C6	1.0860 (7)	0.4090 (7)	0.0742 (6)	0.051 (3)	
H6	1.1582	0.4114	0.0768	0.061*	
C7	1.0393 (7)	0.3399 (6)	0.0463 (5)	0.044 (2)	
H7	1.0799	0.2941	0.0277	0.053*	
C8	0.9346 (7)	0.3315 (6)	0.0429 (6)	0.043 (2)	
C9	0.8779 (8)	0.2534 (6)	0.0144 (6)	0.050 (2)	
C10	0.6997 (7)	0.2185 (6)	−0.0096 (6)	0.046 (2)	
C11	0.5957 (6)	0.2496 (5)	0.0048 (5)	0.0338 (19)	
C12	0.5061 (7)	0.2080 (6)	−0.0132 (6)	0.046 (2)	
H12	0.5073	0.1516	−0.0335	0.055*	
C13	0.4141 (7)	0.2496 (6)	−0.0013 (6)	0.040 (2)	
H13	0.3521	0.2211	−0.0135	0.048*	
C14	0.4115 (6)	0.3322 (6)	0.0283 (5)	0.041 (2)	
H14	0.3485	0.3605	0.0361	0.049*	
C15	0.5029 (6)	0.3725 (6)	0.0463 (6)	0.039 (2)	
H15	0.5024	0.4288	0.0671	0.047*	
C16	0.5319 (7)	0.4149 (5)	0.7455 (5)	0.0369 (19)	
H16A	0.4730	0.4233	0.7804	0.044*	
H16B	0.5941	0.4171	0.7785	0.044*	
C17	0.5240 (7)	0.3253 (6)	0.7090 (6)	0.043 (2)	
H17A	0.4616	0.3203	0.6765	0.052*	
H17B	0.5838	0.3134	0.6758	0.052*	
C18	0.5204 (8)	0.2630 (6)	0.7782 (6)	0.051 (2)	
H18A	0.5767	0.2779	0.8146	0.061*	
H18B	0.4560	0.2730	0.8064	0.061*	
C19	0.5275 (8)	0.1699 (7)	0.7611 (7)	0.058 (3)	
H19A	0.4918	0.1574	0.7119	0.087*	
H19B	0.4962	0.1375	0.8036	0.087*	
H19C	0.5992	0.1535	0.7565	0.087*	
C20	0.4472 (7)	0.4901 (6)	0.6316 (5)	0.043 (2)	
H20A	0.4527	0.5411	0.5977	0.051*	
H20B	0.4512	0.4391	0.5976	0.051*	
C21	0.3459 (7)	0.4911 (6)	0.6706 (6)	0.050 (3)	
H21A	0.3421	0.5405	0.7067	0.060*	
H21B	0.3374	0.4383	0.7017	0.060*	
C22	0.2616 (8)	0.4973 (6)	0.6096 (7)	0.054 (3)	
H22A	0.2663	0.4491	0.5724	0.065*	
H22B	0.2679	0.5513	0.5799	0.065*	
C23	0.1598 (7)	0.4947 (7)	0.6519 (7)	0.057 (3)	
H23A	0.1719	0.4909	0.7084	0.086*	
H23B	0.1209	0.4448	0.6343	0.086*	
H23C	0.1213	0.5468	0.6401	0.086*	
C24	0.5404 (7)	0.5681 (6)	0.7370 (5)	0.041 (2)	
H24A	0.6013	0.5648	0.7713	0.050*	
H24B	0.4798	0.5701	0.7709	0.050*	
C25	0.5454 (7)	0.6510 (6)	0.6889 (6)	0.047 (2)	
H25A	0.6000	0.6466	0.6496	0.057*	
H25B	0.4800	0.6600	0.6609	0.057*	
C26	0.5662 (7)	0.7246 (6)	0.7426 (6)	0.045 (2)	
H26A	0.6334	0.7170	0.7682	0.054*	
H26B	0.5139	0.7266	0.7839	0.054*	
C27	0.5649 (8)	0.8075 (6)	0.6969 (7)	0.054 (3)	
H27A	0.6076	0.8016	0.6505	0.081*	
H27B	0.5912	0.8537	0.7299	0.081*	
H27C	0.4949	0.8207	0.6807	0.081*	
C28	0.6286 (7)	0.4810 (6)	0.6329 (6)	0.043 (2)	
H28A	0.6229	0.4262	0.6044	0.052*	
H28B	0.6246	0.5273	0.5936	0.052*	
C29	0.7327 (7)	0.4846 (6)	0.6714 (6)	0.045 (2)	
H29A	0.7475	0.5434	0.6896	0.054*	
H29B	0.7353	0.4458	0.7171	0.054*	
C30	0.8095 (7)	0.4574 (6)	0.6112 (6)	0.044 (2)	
H30A	0.7961	0.3974	0.5962	0.053*	
H30B	0.8009	0.4931	0.5638	0.053*	
C31	0.9170 (7)	0.4649 (7)	0.6398 (7)	0.054 (3)	
H31D	0.9180	0.4931	0.6909	0.081*	
H31E	0.9565	0.4987	0.6025	0.081*	
H31F	0.9469	0.4078	0.6446	0.081*	
Fe1	0.72938 (9)	0.37769 (8)	0.05494 (8)	0.0383 (4)	
N1	0.7165 (6)	0.4653 (5)	−0.1095 (5)	0.047 (2)	
N2	0.7152 (5)	0.3071 (5)	0.2246 (5)	0.0452 (19)	
N3	0.6753 (6)	0.5540 (6)	0.1211 (5)	0.051 (2)	
N4	0.8754 (5)	0.3996 (5)	0.0672 (4)	0.0392 (17)	
N5	0.7711 (5)	0.2806 (5)	0.0184 (4)	0.0405 (18)	
N6	0.5944 (5)	0.3314 (5)	0.0340 (4)	0.0371 (17)	
N7	0.5353 (5)	0.4892 (4)	0.6877 (4)	0.0375 (17)	
O1	0.9148 (4)	0.1857 (4)	0.0004 (4)	0.0468 (16)	
O2	0.7099 (5)	0.1495 (4)	−0.0387 (4)	0.0491 (17)	
O1W	0.8661 (5)	0.8573 (5)	0.0106 (4)	0.061 (2)	
H1X	0.9274	0.8405	0.0077	0.073*	
H1Y	0.8287	0.8276	0.0404	0.073*	
O2W	0.7877 (5)	0.7228 (4)	0.1248 (4)	0.0450 (16)	
H2X	0.7868	0.7474	0.1691	0.054*	
H2Y	0.7576	0.6750	0.1200	0.054*	

### Atomic displacement parameters (Å^2^)

**Table:**

	*U*^11^	*U*^22^	*U*^33^	*U*^12^	*U*^13^	*U*^23^
C1	0.043 (5)	0.047 (5)	0.032 (5)	−0.018 (4)	0.000 (4)	0.010 (4)
C2	0.042 (5)	0.041 (5)	0.035 (5)	−0.002 (4)	−0.006 (4)	0.008 (4)
C3	0.051 (5)	0.038 (5)	0.039 (5)	−0.002 (4)	−0.009 (4)	0.019 (4)
C4	0.041 (5)	0.049 (5)	0.047 (6)	−0.007 (4)	0.001 (4)	0.023 (5)
C5	0.047 (5)	0.053 (6)	0.048 (6)	−0.009 (4)	−0.010 (5)	0.020 (5)
C6	0.038 (5)	0.061 (6)	0.053 (6)	−0.010 (4)	−0.011 (4)	0.018 (5)
C7	0.045 (5)	0.054 (5)	0.032 (5)	−0.003 (4)	−0.005 (4)	0.014 (4)
C8	0.041 (5)	0.042 (5)	0.046 (6)	0.001 (4)	−0.003 (4)	0.022 (4)
C9	0.062 (6)	0.043 (5)	0.044 (6)	0.015 (4)	−0.014 (5)	−0.008 (5)
C10	0.041 (5)	0.039 (5)	0.059 (7)	0.003 (4)	−0.008 (4)	0.011 (5)
C11	0.030 (4)	0.036 (4)	0.036 (5)	−0.001 (3)	−0.003 (3)	0.009 (4)
C12	0.047 (5)	0.043 (5)	0.048 (6)	−0.009 (4)	−0.001 (4)	0.014 (5)
C13	0.037 (5)	0.042 (5)	0.041 (5)	−0.008 (4)	−0.007 (4)	0.019 (4)
C14	0.034 (4)	0.051 (5)	0.037 (5)	0.003 (4)	−0.005 (4)	0.014 (4)
C15	0.035 (4)	0.041 (5)	0.042 (5)	−0.003 (3)	−0.003 (4)	−0.001 (4)
C16	0.046 (5)	0.038 (5)	0.027 (4)	0.014 (4)	0.004 (4)	0.002 (4)
C17	0.051 (5)	0.041 (5)	0.038 (6)	0.007 (4)	0.004 (4)	0.018 (4)
C18	0.055 (6)	0.051 (6)	0.048 (6)	0.002 (4)	0.007 (5)	0.015 (5)
C19	0.070 (7)	0.052 (6)	0.054 (7)	−0.006 (5)	0.029 (5)	0.016 (5)
C20	0.058 (6)	0.043 (5)	0.028 (5)	0.016 (4)	−0.004 (4)	−0.011 (4)
C21	0.058 (6)	0.046 (5)	0.046 (6)	0.021 (4)	−0.012 (5)	−0.020 (5)
C22	0.060 (6)	0.045 (5)	0.057 (7)	0.012 (4)	−0.008 (5)	−0.016 (5)
C23	0.050 (6)	0.055 (6)	0.067 (8)	0.017 (4)	−0.002 (5)	−0.032 (6)
C24	0.053 (5)	0.044 (5)	0.027 (5)	0.001 (4)	0.003 (4)	−0.006 (4)
C25	0.052 (6)	0.048 (5)	0.042 (6)	0.009 (4)	−0.006 (4)	−0.010 (5)
C26	0.047 (5)	0.048 (5)	0.039 (5)	−0.020 (4)	0.005 (4)	−0.014 (4)
C27	0.065 (6)	0.048 (6)	0.048 (6)	−0.006 (4)	−0.020 (5)	−0.005 (5)
C28	0.054 (5)	0.038 (5)	0.037 (5)	0.018 (4)	0.012 (4)	0.007 (4)
C29	0.057 (6)	0.041 (5)	0.038 (5)	0.011 (4)	0.021 (4)	0.010 (4)
C30	0.045 (5)	0.040 (5)	0.048 (6)	0.012 (4)	0.008 (4)	0.001 (4)
C31	0.054 (6)	0.054 (6)	0.054 (7)	0.003 (4)	0.012 (5)	0.017 (5)
Fe1	0.0407 (7)	0.0414 (8)	0.0326 (7)	−0.0164 (5)	−0.0082 (5)	0.0165 (6)
N1	0.045 (4)	0.047 (4)	0.048 (5)	−0.021 (3)	0.000 (4)	0.014 (4)
N2	0.041 (4)	0.054 (5)	0.041 (5)	−0.006 (3)	−0.004 (3)	0.011 (4)
N3	0.055 (5)	0.054 (5)	0.045 (5)	0.015 (4)	−0.005 (4)	0.014 (4)
N4	0.037 (4)	0.049 (4)	0.032 (4)	−0.010 (3)	−0.003 (3)	0.012 (3)
N5	0.037 (4)	0.054 (4)	0.031 (4)	−0.010 (3)	−0.006 (3)	0.023 (4)
N6	0.038 (4)	0.041 (4)	0.032 (4)	−0.002 (3)	−0.002 (3)	0.014 (3)
N7	0.046 (4)	0.041 (4)	0.026 (4)	0.008 (3)	−0.006 (3)	−0.008 (3)
O1	0.042 (3)	0.051 (4)	0.047 (4)	0.014 (3)	−0.002 (3)	−0.012 (3)
O2	0.052 (4)	0.045 (4)	0.050 (4)	0.017 (3)	−0.008 (3)	0.002 (3)
O1W	0.065 (5)	0.066 (5)	0.052 (5)	−0.026 (3)	−0.007 (4)	−0.020 (4)
O2W	0.053 (4)	0.044 (3)	0.038 (4)	−0.019 (3)	0.012 (3)	−0.015 (3)

### Geometric parameters (Å, °)

**Table:**

C1—N1	1.195 (12)	C20—N7	1.497 (11)
C1—Fe1	1.940 (9)	C20—H20A	0.9900
C2—N2	1.146 (11)	C20—H20B	0.9900
C2—Fe1	1.966 (10)	C21—C22	1.518 (14)
C3—N3	1.140 (12)	C21—H21A	0.9900
C3—Fe1	1.933 (11)	C21—H21B	0.9900
C4—C5	1.383 (13)	C22—C23	1.526 (15)
C4—N4	1.391 (12)	C22—H22A	0.9900
C4—H4	0.9500	C22—H22B	0.9900
C5—C6	1.379 (15)	C23—H23A	0.9800
C5—H5	0.9500	C23—H23B	0.9800
C6—C7	1.331 (14)	C23—H23C	0.9800
C6—H6	0.9500	C24—N7	1.497 (11)
C7—C8	1.383 (12)	C24—C25	1.539 (14)
C7—H7	0.9500	C24—H24A	0.9900
C8—N4	1.386 (12)	C24—H24B	0.9900
C8—C9	1.511 (14)	C25—C26	1.499 (12)
C9—O1	1.192 (11)	C25—H25A	0.9900
C9—N5	1.470 (12)	C25—H25B	0.9900
C10—O2	1.197 (11)	C26—C27	1.515 (14)
C10—N5	1.431 (12)	C26—H26A	0.9900
C10—C11	1.474 (12)	C26—H26B	0.9900
C11—N6	1.375 (11)	C27—H27A	0.9800
C11—C12	1.378 (12)	C27—H27B	0.9800
C12—C13	1.390 (13)	C27—H27C	0.9800
C12—H12	0.9500	C28—C29	1.515 (14)
C13—C14	1.390 (13)	C28—N7	1.555 (11)
C13—H13	0.9500	C28—H28A	0.9900
C14—C15	1.389 (12)	C28—H28B	0.9900
C14—H14	0.9500	C29—C30	1.510 (12)
C15—N6	1.383 (11)	C29—H29A	0.9900
C15—H15	0.9500	C29—H29B	0.9900
C16—N7	1.528 (11)	C30—C31	1.495 (14)
C16—C17	1.540 (13)	C30—H30A	0.9900
C16—H16A	0.9900	C30—H30B	0.9900
C16—H16B	0.9900	C31—H31D	0.9800
C17—C18	1.536 (13)	C31—H31E	0.9800
C17—H17A	0.9900	C31—H31F	0.9800
C17—H17B	0.9900	Fe1—N5	1.734 (8)
C18—C19	1.490 (15)	Fe1—N6	1.946 (7)
C18—H18A	0.9900	Fe1—N4	1.959 (7)
C18—H18B	0.9900	O1W—H1X	0.8500
C19—H19A	0.9800	O1W—H1Y	0.8500
C19—H19B	0.9800	O2W—H2X	0.8502
C19—H19C	0.9800	O2W—H2Y	0.8499
C20—C21	1.494 (14)		
			
N1—C1—Fe1	174.6 (8)	C22—C23—H23C	109.5
N2—C2—Fe1	176.4 (8)	H23A—C23—H23C	109.5
N3—C3—Fe1	174.6 (8)	H23B—C23—H23C	109.5
C5—C4—N4	120.3 (9)	N7—C24—C25	113.4 (7)
C5—C4—H4	119.9	N7—C24—H24A	108.9
N4—C4—H4	119.9	C25—C24—H24A	108.9
C6—C5—C4	120.0 (9)	N7—C24—H24B	108.9
C6—C5—H5	120.0	C25—C24—H24B	108.9
C4—C5—H5	120.0	H24A—C24—H24B	107.7
C7—C6—C5	119.1 (9)	C26—C25—C24	109.3 (8)
C7—C6—H6	120.4	C26—C25—H25A	109.8
C5—C6—H6	120.4	C24—C25—H25A	109.8
C6—C7—C8	123.2 (10)	C26—C25—H25B	109.8
C6—C7—H7	118.4	C24—C25—H25B	109.8
C8—C7—H7	118.4	H25A—C25—H25B	108.3
C7—C8—N4	118.4 (8)	C25—C26—C27	110.0 (8)
C7—C8—C9	125.3 (9)	C25—C26—H26A	109.7
N4—C8—C9	116.3 (8)	C27—C26—H26A	109.7
O1—C9—N5	131.2 (9)	C25—C26—H26B	109.7
O1—C9—C8	125.8 (9)	C27—C26—H26B	109.7
N5—C9—C8	102.6 (7)	H26A—C26—H26B	108.2
O2—C10—N5	132.7 (9)	C26—C27—H27A	109.5
O2—C10—C11	118.3 (8)	C26—C27—H27B	109.5
N5—C10—C11	109.0 (8)	H27A—C27—H27B	109.5
N6—C11—C12	120.6 (8)	C26—C27—H27C	109.5
N6—C11—C10	112.5 (7)	H27A—C27—H27C	109.5
C12—C11—C10	126.7 (9)	H27B—C27—H27C	109.5
C11—C12—C13	119.3 (9)	C29—C28—N7	116.7 (8)
C11—C12—H12	120.4	C29—C28—H28A	108.1
C13—C12—H12	120.4	N7—C28—H28A	108.1
C14—C13—C12	120.8 (8)	C29—C28—H28B	108.1
C14—C13—H13	119.6	N7—C28—H28B	108.1
C12—C13—H13	119.6	H28A—C28—H28B	107.3
C15—C14—C13	118.7 (8)	C30—C29—C28	107.4 (8)
C15—C14—H14	120.6	C30—C29—H29A	110.2
C13—C14—H14	120.6	C28—C29—H29A	110.2
N6—C15—C14	120.4 (8)	C30—C29—H29B	110.2
N6—C15—H15	119.8	C28—C29—H29B	110.2
C14—C15—H15	119.8	H29A—C29—H29B	108.5
N7—C16—C17	115.7 (7)	C31—C30—C29	112.9 (9)
N7—C16—H16A	108.4	C31—C30—H30A	109.0
C17—C16—H16A	108.4	C29—C30—H30A	109.0
N7—C16—H16B	108.4	C31—C30—H30B	109.0
C17—C16—H16B	108.4	C29—C30—H30B	109.0
H16A—C16—H16B	107.4	H30A—C30—H30B	107.8
C18—C17—C16	105.6 (8)	C30—C31—H31D	109.5
C18—C17—H17A	110.6	C30—C31—H31E	109.5
C16—C17—H17A	110.6	H31D—C31—H31E	109.5
C18—C17—H17B	110.6	C30—C31—H31F	109.5
C16—C17—H17B	110.6	H31D—C31—H31F	109.5
H17A—C17—H17B	108.8	H31E—C31—H31F	109.5
C19—C18—C17	117.9 (9)	N5—Fe1—C3	176.6 (4)
C19—C18—H18A	107.8	N5—Fe1—C1	96.1 (4)
C17—C18—H18A	107.8	C3—Fe1—C1	82.9 (4)
C19—C18—H18B	107.8	N5—Fe1—N6	84.1 (3)
C17—C18—H18B	107.8	C3—Fe1—N6	99.1 (4)
H18A—C18—H18B	107.2	C1—Fe1—N6	88.7 (3)
C18—C19—H19A	109.5	N5—Fe1—N4	83.1 (3)
C18—C19—H19B	109.5	C3—Fe1—N4	93.6 (4)
H19A—C19—H19B	109.5	C1—Fe1—N4	92.9 (3)
C18—C19—H19C	109.5	N6—Fe1—N4	167.2 (3)
H19A—C19—H19C	109.5	N5—Fe1—C2	92.4 (4)
H19B—C19—H19C	109.5	C3—Fe1—C2	88.8 (4)
C21—C20—N7	113.7 (8)	C1—Fe1—C2	171.2 (4)
C21—C20—H20A	108.8	N6—Fe1—C2	90.0 (3)
N7—C20—H20A	108.8	N4—Fe1—C2	90.3 (3)
C21—C20—H20B	108.8	C8—N4—C4	119.0 (7)
N7—C20—H20B	108.8	C8—N4—Fe1	112.6 (6)
H20A—C20—H20B	107.7	C4—N4—Fe1	128.4 (6)
C20—C21—C22	110.0 (9)	C10—N5—C9	114.2 (8)
C20—C21—H21A	109.7	C10—N5—Fe1	120.6 (6)
C22—C21—H21A	109.7	C9—N5—Fe1	125.2 (6)
C20—C21—H21B	109.7	C11—N6—C15	120.1 (7)
C22—C21—H21B	109.7	C11—N6—Fe1	113.6 (5)
H21A—C21—H21B	108.2	C15—N6—Fe1	126.3 (6)
C21—C22—C23	108.1 (9)	C24—N7—C20	112.6 (6)
C21—C22—H22A	110.1	C24—N7—C16	105.4 (7)
C23—C22—H22A	110.1	C20—N7—C16	113.3 (7)
C21—C22—H22B	110.1	C24—N7—C28	112.0 (7)
C23—C22—H22B	110.1	C20—N7—C28	103.0 (7)
H22A—C22—H22B	108.4	C16—N7—C28	110.7 (6)
C22—C23—H23A	109.5	H1X—O1W—H1Y	114.6
C22—C23—H23B	109.5	H2X—O2W—H2Y	118.5
H23A—C23—H23B	109.5		
			
N4—C4—C5—C6	−0.5 (14)	C11—C10—N5—C9	174.3 (8)
C4—C5—C6—C7	0.8 (15)	O2—C10—N5—Fe1	177.8 (10)
C5—C6—C7—C8	−1.8 (15)	C11—C10—N5—Fe1	−4.6 (10)
C6—C7—C8—N4	2.3 (14)	O1—C9—N5—C10	−9.8 (15)
C6—C7—C8—C9	−177.7 (9)	C8—C9—N5—C10	177.0 (7)
C7—C8—C9—O1	9.8 (17)	O1—C9—N5—Fe1	169.0 (9)
N4—C8—C9—O1	−170.2 (10)	C8—C9—N5—Fe1	−4.1 (10)
C7—C8—C9—N5	−176.5 (9)	C1—Fe1—N5—C10	−86.2 (7)
N4—C8—C9—N5	3.4 (11)	N6—Fe1—N5—C10	1.8 (7)
O2—C10—C11—N6	−176.4 (9)	N4—Fe1—N5—C10	−178.4 (7)
N5—C10—C11—N6	5.5 (11)	C2—Fe1—N5—C10	91.6 (7)
O2—C10—C11—C12	−1.4 (15)	C1—Fe1—N5—C9	95.0 (8)
N5—C10—C11—C12	−179.5 (8)	N6—Fe1—N5—C9	−176.9 (8)
N6—C11—C12—C13	−0.2 (13)	N4—Fe1—N5—C9	2.8 (7)
C10—C11—C12—C13	−174.8 (9)	C2—Fe1—N5—C9	−87.2 (8)
C11—C12—C13—C14	0.1 (14)	C12—C11—N6—C15	0.5 (12)
C12—C13—C14—C15	−0.3 (13)	C10—C11—N6—C15	175.9 (8)
C13—C14—C15—N6	0.7 (13)	C12—C11—N6—Fe1	−179.8 (7)
N7—C16—C17—C18	−179.2 (7)	C10—C11—N6—Fe1	−4.5 (9)
C16—C17—C18—C19	−171.5 (8)	C14—C15—N6—C11	−0.8 (13)
N7—C20—C21—C22	176.7 (7)	C14—C15—N6—Fe1	179.6 (6)
C20—C21—C22—C23	177.9 (8)	N5—Fe1—N6—C11	1.7 (6)
N7—C24—C25—C26	−171.5 (7)	C3—Fe1—N6—C11	−179.5 (6)
C24—C25—C26—C27	−176.6 (8)	C1—Fe1—N6—C11	97.9 (6)
N7—C28—C29—C30	168.5 (7)	N4—Fe1—N6—C11	0.5 (17)
C28—C29—C30—C31	175.3 (7)	C2—Fe1—N6—C11	−90.7 (6)
C7—C8—N4—C4	−1.9 (12)	N5—Fe1—N6—C15	−178.7 (8)
C9—C8—N4—C4	178.2 (8)	C3—Fe1—N6—C15	0.2 (8)
C7—C8—N4—Fe1	178.1 (7)	C1—Fe1—N6—C15	−82.4 (8)
C9—C8—N4—Fe1	−1.9 (10)	N4—Fe1—N6—C15	−179.9 (12)
C5—C4—N4—C8	1.1 (13)	C2—Fe1—N6—C15	88.9 (7)
C5—C4—N4—Fe1	−178.9 (7)	C25—C24—N7—C20	−56.5 (10)
N5—Fe1—N4—C8	−0.3 (6)	C25—C24—N7—C16	179.5 (7)
C3—Fe1—N4—C8	−179.2 (6)	C25—C24—N7—C28	59.0 (9)
C1—Fe1—N4—C8	−96.1 (7)	C21—C20—N7—C24	−63.4 (10)
N6—Fe1—N4—C8	0.8 (17)	C21—C20—N7—C16	56.0 (9)
C2—Fe1—N4—C8	92.0 (6)	C21—C20—N7—C28	175.7 (7)
N5—Fe1—N4—C4	179.6 (8)	C17—C16—N7—C24	178.7 (7)
C3—Fe1—N4—C4	0.7 (8)	C17—C16—N7—C20	55.2 (9)
C1—Fe1—N4—C4	83.8 (8)	C17—C16—N7—C28	−59.9 (9)
N6—Fe1—N4—C4	−179.2 (12)	C29—C28—N7—C24	54.5 (10)
C2—Fe1—N4—C4	−88.0 (8)	C29—C28—N7—C20	175.7 (7)
O2—C10—N5—C9	−3.4 (15)	C29—C28—N7—C16	−62.8 (9)

### Hydrogen-bond geometry (Å, °)

**Table:**

*D*—H···*A*	*D*—H	H···*A*	*D*···*A*	*D*—H···*A*
O1W—H1X···O1^i^	0.85	2.12	2.964 (10)	173
O1W—H1Y···O2W	0.85	2.25	3.058 (10)	158
O2W—H2X···N2^ii^	0.85	2.04	2.895 (10)	180
O2W—H2Y···N3	0.85	2.18	3.029 (11)	174

Symmetry codes: (i) −*x*+2, −*y*+1, −*z*; (ii) −*x*+3/2, *y*+1/2, −*z*+1/2.
